# Effectiveness of BNT162b2 Vaccine for Preventing COVID-19-Related Hospitalizations: A Test-Negative Case–Control Study

**DOI:** 10.3390/vaccines12060657

**Published:** 2024-06-13

**Authors:** Amy Keane, Ashley Tippett, Elizabeth Grace Taylor, Olivia Reese, Luis Salazar, Khalel De Castro, Chris Choi, Caroline Ciric, Meg Taylor, Anna Mitchell, Theda Gibson, Laura Puzniak, Robin Hubler, Srinivas Rao Valluri, Timothy L. Wiemken, Ben A. Lopman, Satoshi Kamidani, Larry J. Anderson, John M. McLaughlin, Christina A. Rostad, Evan J. Anderson

**Affiliations:** 1 Department of Pediatrics, Emory University School of Medicine, Atlanta, GA 30322, USA; amyekeane@gmail.com (A.K.); ashley.tippett@emory.edu (A.T.); elizabeth.grace.taylor@emory.edu (E.G.T.); olivia.day.reese@emory.edu (O.R.); luis.w.salazar@emory.edu (L.S.); khalel.de.castro@emory.edu (K.D.C.); chris.choi@emory.edu (C.C.); caroline.ciric@emory.edu (C.C.); meg96taylor@gmail.com (M.T.); akm20a@fsu.edu (A.M.); theda.gibson@emory.edu (T.G.); larry.anderson@emory.edu (L.J.A.); evanderson@emory.edu (E.J.A.); 2 Pfizer, Inc., New York, NY 10017, USA; laura.puzniak@pfizer.com (L.P.); robin.hubler@pfizer.com (R.H.); srinivas.rao.valluri@pfizer.com (S.R.V.); timothy.wiemken@pfizer.com (T.L.W.); john.mclaughlin@pfizer.com (J.M.M.); 3 Rollins School of Public Health, Emory University, Atlanta, GA 30322, USA; benjamin.alan.lopman@emory.edu; 4 Center for Childhood Infections and Vaccines, Children’s Healthcare of Atlanta, Atlanta, GA 30322, USA; 5 Division of Infectious Diseases, Department of Medicine, Emory University School of Medicine, Atlanta, GA 30322, USA

**Keywords:** SARS-CoV-2, hospital, vaccine, variant, Omicron, Delta, vaccine effectiveness

## Abstract

It is important to understand real-world BNT162b2 COVID-19 vaccine effectiveness (VE), especially among racial and ethnic minority groups. We performed a test-negative case-control study to measure BNT162b2 COVID-19 VE in the prevention of COVID-19-associated acute respiratory illness (ARI) hospitalizations at two Atlanta hospitals from May 2021–January 2023 and adjusted for potential confounders by multivariate analysis. Among 5139 eligible adults with ARI, 2763 (53.8%) were enrolled, and 1571 (64.5%) were included in the BNT162b2 analysis. The median age was 58 years (IQR, 44–68), 889 (56.6%) were female, 1034 (65.8%) were African American, 359 (22.9%) were White, 56 (3.6%) were Hispanic ethnicity, 645 (41.1%) were SARS-CoV-2-positive, 412 (26.2%) were vaccinated with a primary series, and 273 (17.4%) had received ≥1 booster of BNT162b2. The overall adjusted VE of the BNT162b2 primary series was 58.5% (95% CI 46.0, 68.1), while the adjusted VE of ≥1 booster was 78.9% (95% CI 70.0, 85.1). The adjusted overall VE of primary series for African American/Black individuals was 64.0% (95% CI 49.9, 74.1) and 82.7% (95% CI 71.9, 89.4) in those who received ≥1 booster. When analysis was limited to the period of Omicron predominance, overall VE of the primary series decreased with widened confidence intervals (24.5%, 95% CI −4.5, 45.4%), while VE of ≥1 booster was maintained at 60.9% (95% CI 42.0, 73.6). BNT162b2 primary series and booster vaccination provided protection against COVID-19-associated ARI hospitalization among a predominantly African American population.

## 1. Introduction

The severe acute respiratory syndrome coronavirus 2 (SARS-CoV-2), which causes coronavirus disease 2019 (COVID-19), has resulted in over 6.5 million hospitalizations and 1.1 million deaths in the U.S. as of December 2023 [1]. The rapid development, authorization, and administration of COVID-19 vaccinations mitigated the impact of the virus, averting an estimated 18.6 million hospitalizations and 3.2 million deaths in the first two years of administration from 12 December 2020 through 30 November 2022 alone [2]. The majority of COVID-19 vaccines administered in the United States, including BNT162b2 (Pfizer–BioNTech) and mRNA-1273 (Moderna, Inc.), were based on novel mRNA technology, which facilitated rapid design, testing, and scalable mass production of vaccines. As of 10 May 2023, 81% of the U.S. population had received at least one dose of the COVID-19 vaccination, including more than 400 million doses of BNT162b2 [1]. However, racial and ethnic minority groups have lower uptake compared to White, non-Hispanic individuals [3].

BNT162b2 is a lipid nanoparticle-formulated nucleoside-modified mRNA encoding the pre-fusion-stabilized SARS-CoV-2 full-length spike protein [4]. In the phase 3 randomized controlled pivotal trial, vaccination with the BNT162b2 mRNA vaccine conferred 95% efficacy in the prevention of ancestral SARS-CoV-2 infections (e.g., Wa-1, D614G strains) in adults [5]. Based on these data, BNT162b2 received emergency use authorization (EUA) on 11 December 2020 and later became fully licensed on 23 August 2021 for individuals 16 years of age and older. The emergence of the Delta variant prompted the recommendation for a third dose of mRNA COVID-19 vaccines in the fall of 2021. The subsequent emergence of the Omicron variant underscored the importance of booster doses and prompted the recommendation of bivalent COVID-19 vaccines (containing equal amounts of mRNA encoding the ancestral and Omicron BA.4/BA.5 strains) in August 2022 [6]. As Omicron subvariants such as XBB.1.5 became predominant in 2023, in September 2023, the Centers for Disease Control (CDC) recommended the administration of an updated monovalent XBB.1.5-containing booster [7].

The emergence of SARS-CoV-2 variants and evolving population-based immunity underscore the need for ongoing assessments of real-world COVID-19 vaccine effectiveness. Although the initial clinical trials of BNT162b2 provided strong evidence of vaccine efficacy, real-world VE may be impacted by multiple factors, including heterogeneity in the patient population with more underlying comorbidities, hybrid immunity, waning of immune responses, emergence of new variants of concern, and updated booster recommendations [8,9,10,11]. Further, racial and ethnic minority groups report less COVID-19 vaccine confidence and lower vaccination rates, despite having the highest risk of morbidity and mortality in the United States. There are limited vaccine effectiveness data evaluating racial and ethnic minority populations. Developing evidence specific to these populations may provide additional reassurance on the value of vaccination and responses to vaccine hesitancy among minority groups to help reduce the burden of disease [3,12,13]. The overall objective of this study was to determine the real-world effectiveness of the BNT162b2 vaccination in preventing COVID-19-related hospitalization in adults using a test-negative case–control design among a predominantly African American/Black population.

## 2. Materials and Methods

### 2.1. Study Enrollment

This study was reviewed and approved by the Institutional Review Board at Emory University. Between the dates of 2 May 2021 and 31 January 2023, adults admitted to Emory University Hospital (EUH) and Emory University Hospital Midtown (EUHM) with acute respiratory illness (ARI) were approached to consent and enroll. Individuals were eligible to enroll if they were ≥18 years of age, presented to the hospital with ARI symptoms (defined as nasal congestion, rhinorrhea, sore throat, hoarseness, new or increased-from-baseline cough, sputum production, dyspnea, wheezing), or had an admitting diagnosis suggestive of ARI (pneumonia, upper respiratory infection, bronchitis, influenza, cough, asthma, viral respiratory illness, respiratory distress, or respiratory failure). Participants could not have previously enrolled in the study within the past 30 days. Patients were excluded if they had received SARS-CoV-2-directed antiviral treatment within the preceding 30 days, had received COVID-19 monoclonal antibody or convalescent serum therapies within the preceding 90 days, if they had received any dose of a non-BNT162b2 COVID-19 vaccine, or if they did not have a SARS-CoV-2 test result available. Sociodemographic and clinical characteristics were collected through medical record abstraction and an in-depth patient interview, unless this was not possible due to an underlying medical condition.

### 2.2. Laboratory Testing

Beginning in the summer of 2020, nearly all adults admitted to these hospitals received standard-of-care (SOC) testing for SARS-CoV-2. This provided a unique opportunity to evaluate the VE of the BNT162b2 vaccination against hospitalization due to ARI. Cases and controls were primarily identified using the results of SOC testing. This process involved the collection of a nasopharyngeal (NP) swab, which was tested for the presence of SARS-CoV-2 by nucleic acid amplification testing (either GeneXpert (Cepheid, Sunnyvale, CA, USA) or BioFire (bioMerieux, Inc., Salt Lake City, UT, USA) respiratory panel) in the clinical laboratories. If a patient did not have an SOC specimen but had documentation of a positive outside COVID-19 test, they were also classified as a case.

### 2.3. Vaccine Verification and Vaccination Status Definitions

We determined the vaccination status of each patient through a review of the Georgia Registry of Immunization Transactions and Services and the electronic medical record. These data were used to classify each participant into one of the three pre-defined exposure groups based on the number of doses received and their immunocompetency status. Inclusion in the exposure groups required receipt of the most recent vaccination ≥14 days prior to hospitalization. Completion of a primary series was defined as the receipt of two BNT162b2 doses among immunocompetent individuals and three BNT162b2 doses among immunocompromised individuals (either an ancestral strain or BA.4/5 bivalent). Immunocompetent individuals were considered boosted if they had ≥3 BNT162b2 doses or ≥4 BNT162b2 doses if they were immunocompromised. Immunocompromised status was defined as participants who had been formally diagnosed with HIV/AIDS, any type of rheumatologic condition/connective tissue disease, active cancer, history of hematopoietic cell or solid organ transplant, history of splenectomy, or those who had been taking steroids for ≥2 weeks at the time of hospitalization. The unvaccinated group was defined as not having received BNT162b2 or any other COVID-19 vaccine at the time of hospital admission and served as the reference ‘unexposed’ group in all VE analyses. Patients whose vaccination status did not align with any of these three groups were excluded from this analysis. Hybrid immunity was defined as having received the BNT162b2 vaccination as above and self-reporting a prior history of SARS-CoV-2 infection.

### 2.4. Study Outcomes

The objectives of this study were to determine the odds of being vaccinated with a primary series or ≥1 booster of BNT162b2 among cases and test-negative controls. VE estimates of the primary series and booster (inclusive of either ancestral strain or BA.4/5 bivalent vaccine) were stratified by African American/Black race, White race, era of SARS-CoV-2 variant circulation, and time since the last BNT162b2 dose (<6 months vs. ≥6 months). For the purposes of this analysis, the time period from 2 May 2021 to 19 December 2021 was classified as the pre-Delta/Delta era, and the period from 20 December 2021 to 31 January 2023 was classified as the Omicron era based on the time the Omicron BA.1 variant became predominant in the U.S. [14]. Although the sample size was limited, the VE of the BA.4/5 bivalent vaccine was determined as a subgroup analysis (Supplementary Results). This study concluded before the XBB.1.5 monovalent vaccine became available.

### 2.5. Statistical Analysis

Sociodemographic and clinical characteristics were assessed using bivariate analysis, with a two-tailed *p*-value of <0.05 being considered statistically significant. *T*-tests, Χ^2^, or Fisher exact tests were used where appropriate. We estimated crude VE by constructing and comparing odds ratios (ORs) and 95% confidence intervals (CIs) for having received BNT162b2 (primary series vaccination or booster) for cases and test-negative controls. VE was calculated as 1-OR multiplied by 100%, and corresponding 95% CIs were calculated using the Wald method. In addition to the construction of crude OR and VE estimates, stepwise multivariable logistic regression analysis was performed to control for age, sex, race/ethnicity, and immunocompromised status. This model was used to create an adjusted VE for each exposure group. Statistical analyses were performed using SAS version 9.4 software.

## 3. Results

### 3.1. Study Population

**Figure 1:**
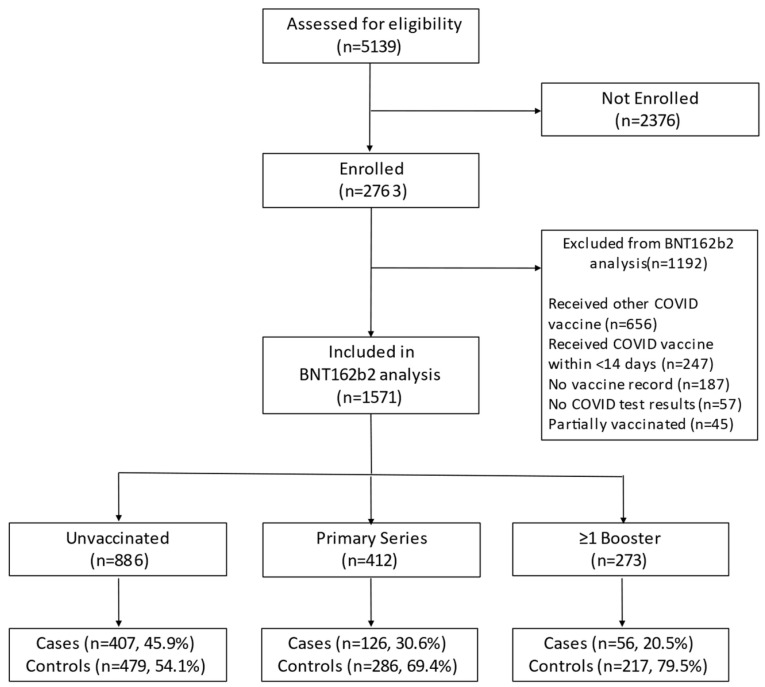
Study enrollment.

Among 5139 potentially eligible adults, 2763 (53.8%) were enrolled, of whom 1571 (56.9%) were eligible for inclusion in the BNT162b2 analysis (Figure 1, Supplementary Results).

The most common reasons for non-enrollment were refusal by the patient or legally authorized representative (64.6%) or discharge prior to being approached by study staff (23.8%). The most common reason for exclusion from analysis after enrollment was receipt of a SARS-CoV-2 vaccine other than BNT162b2 (55.0%). Of the 1571 patients eligible for analyses, the median age was 58 years (IQR, 44–68). Participants who identified as Black/African American comprised the majority (65.8%) of the population, followed by Whites (22.9%), multiracials (2.5%), and other races (1.4%). The majority of participants identified as non-Hispanic (86.4%), and 56 (3.6%) identified as Hispanic. The most common comorbidities observed in the population were hypertension (59.2%), obesity (43.2%), any immunosuppressive condition (31.5%), diabetes mellitus (28.5%), congestive heart failure (23.7%), and chronic kidney disease (21.9%) (Table 1).

### 3.2. Cases vs. Controls

Of the 1571 eligible participants, 645 (41.1%) had SARS-CoV-2 detected and were classified as cases, while 926 (58.9%) tested negative for SARS-CoV-2 and were classified as controls. The majority of cases were enrolled during the pre-Delta/Delta era (2 May 2021–19 December 2021) (52.7%), while the majority of controls were enrolled during the era of Omicron predominance (20 December 2021–31 January 2023) (79.4%).

Cases were significantly younger than controls (median years [IQR], 54 [41, 66] vs. 60 [47, 70], *p* = 0.0001). There were also significant differences in Hispanic ethnicity (*p* = 0.047), types of insurance (*p* < 0.0001), educational background (*p* < 0.0001), and employment status (*p* < 0.0001) between cases and controls (Table 1). Cases also had a higher overall level of educational attainment and a higher rate of employment than controls (Table 1). In terms of social and behavioral risk factors, cases were more likely to have children <18 years of age residing in the home (32.2% vs. 21.2%, *p* < 0.0001) and to have traveled out of state in the past month (17.4% vs. 8.5%, *p* < 0.0001) (Table 2). Cases also reported more commonly adhering to social distancing (*p* < 0.0001) and masking in public (*p* < 0.0001, indoors; *p* < 0.0001, outdoors), although this was in the context of more frequent travel and employment outside of the home. Compared to controls, cases had a similar frequency of alcohol use (36.4% vs. 33.1%, *p* = 0.0109) and a lower proportion who reported smoking (34.7% vs. 49.1%, *p* < 0.0001).

In terms of underlying comorbidities, cases were more likely to have class 1–3 obesity than controls (47.4% vs. 40.3%, *p* < 0.0001). In contrast, controls more commonly had comorbidities of cardiac disease (71.6% vs. 58.1%, *p* < 0.0001), chronic respiratory disease (42.3% vs. 25.0%, *p* < 0.0001), and especially chronic obstructive pulmonary disease (COPD) (25.9% vs. 11.5%, *p* < 0.0001), use of home supplemental oxygen (13.9% vs. 4%, *p* < 0.0001), chronic kidney disease (24.0% vs. 18.9%, *p* = 0.0171), diabetes mellitus (30.5% vs. 25.7%, *p* = 0.0416), and any immunosuppressive condition (36.0% vs. 25.1%, *p* < 0.0001), including cancer (17.3% vs. 8.2%, *p* < 0.0001). Cases were less likely to have had a prior SARS-CoV-2 infection preceding the index hospitalization (8.7% vs. 17.9%, *p* < 0.0001). There were no significant differences in outcomes between cases and controls, including ICU admission (*p* = 0.2), requirement for mechanical ventilation (*p* = 0.5), and hospital disposition (*p* = 0.4).

### 3.3. Vaccinated vs. Unvaccinated

Of the 1571 eligible participants, 886 (56.4%) had never received a COVID-19 vaccine, 412 (26.2%) had received a primary series of BNT162b2, and 273 (17.1%) had received a primary series plus ≥1 booster of BNT162b2. The majority of vaccinated and boosted participants were enrolled in the later months of the study period (median days [IQR] from the start of the enrollment period: 396 [239, 540] and 527 [470, 585], respectively), during the era of Omicron predominance. Vaccinated participants were significantly older than unvaccinated participants (median years [IQR], primary series: 63 [53, 72] vs. 52 [38, 62]; and boosted: 68 [58, 76] vs. 52 [38, 62], *p* < 0.0001 for both comparisons). There were also significant differences in racial and ethnic distribution among the groups, with a lower proportion of African American participants (primary series: 59.7% vs. 72.7%; and boosted: 52.7% vs. 72.7%; *p* < 0.0001 for both comparisons) and Hispanic participants (primary series: 1.9% vs. 4.6%, *p* = 0.0341; boosted: 2.5% vs. 4.6%, *p* = 0.0005) in the primary series vaccinated and boosted vs. unvaccinated groups. There were also significant differences in types of insurance, education levels, and employment status among vaccinated and unvaccinated participants. The majority of participants who resided in skilled nursing or long-term care facilities had been vaccinated with a primary series or boosted.

In regards to social and behavioral risk factors, primary series vaccinated and boosted participants less commonly had children < 18 years of age residing in the home compared to unvaccinated participants (primary series: 68, 16.5% vs. 300, 33.9%; and boosted: 17, 6.2% vs. 300, 33.9%, *p* < 0.0001 for both comparisons). Primary series vaccinated and boosted participants less commonly reported smoking than unvaccinated participants (primary series: 35, 8.5% vs. 155, 17.5%, *p* < 0.0001; and boosted: 16, 5.9% vs. 155, 17.5%, *p* < 0.0001, respectively). While there were statistically significant differences between boosted and unvaccinated participants in terms of adherence to social distancing and masking precautions, the numerical differences were small and of uncertain clinical significance.

The primary series vaccinated more commonly had underlying comorbidities compared to the unvaccinated group (Table 3). Primary series vaccinated participants more commonly had COPD (35.0% vs. 15.8%, *p* < 0.0001), use of home oxygen (12.6% vs. 7.6%, *p* < 0.0001), arrhythmia (18.7% vs. 10.0%, *p* < 0.0001), coronary artery disease (14.6% vs. 10.2%, *p* = 0.0209), congestive heart failure (26.5% vs. 20.3%, *p* = 0.0133), hypertension (66.7% vs. 50.8%, *p* < 0.0001), chronic kidney disease (26.5% vs. 16.6%, *p* < 0.0001), any immunosuppression (41.7% vs. 28.7%, *p* < 0.0001), cancer (21.8% vs. 9.7%, *p* < 0.0001), or chronic steroid use (17.2% vs. 11.3%, *p* = 0.0032). Similar differences were observed between unvaccinated and boosted participants.

### 3.4. Vaccine Effectiveness Analysis

Of those enrolled, 1298 patients were eligible for the primary series VE analysis, of whom 589 (45.4%) tested positive for SARS-CoV-2, and 412 (31.7%) were vaccinated with a primary series of BNT162b2. After adjustment for potential confounding variables (e.g., age, sex, race/ethnicity, and immunocompromising conditions), the overall adjusted vaccine effectiveness of a primary series of BNT162b2 against hospitalization due to SARS-CoV-2 ARI among individuals ≥18 years of age was 58.5% (95% CI 46.0, 68.1) (Table 4). Adjusted VE was 54.7% (95% CI 32.4, 69.6) within 6 months of completion of the primary series and 61.5% (95% CI 47.7, 71.7) ≥6 months after the primary series.

Of those enrolled, 1159 patients were eligible for the VE analysis of the booster group: 519 (44.8%) tested positive for SARS-CoV-2, and 273 (23.6%) were vaccinated with ≥1 booster dose of BNT162b2. After adjusting for potential confounding variables as above, the overall VE of any BNT162b2 booster vaccination against hospitalization due to SARS-CoV-2 ARI among individuals ≥18 years of age was 78.9% (95% CI 70.0, 85.1). Adjusted VE was 79.3% (95% CI 67.4, 86.8) within 6 months of administration of the booster dose and 77.8% (95% CI 64.2, 86.2) ≥6 months after the booster.

Adjusted VE of a primary series of BNT162b2 against hospitalization due to ARI during the pre-variant/Delta era was 84.4% (95% CI 70.7, 91.6) within 6 months of completion of the primary series and 74.7% (95% CI 44.5, 88.4) ≥6 months after the primary series. VE of a primary series during the Omicron era was −8.9% (95% CI −90.0, 37.6) within 6 months of completion of the primary series and 34.0% (95% CI 5.6, 53.8) ≥6 months after the primary series. For the boosted group, the VE of ≥1 booster dose of BNT162b2 against hospitalization due to ARI during the pre-variant/Delta era was 89.9% (95% CI 0.1, 99.0) during the first 6 months following vaccination and not estimable ≥6 months after vaccination. VE of a ≥1 booster dose of BNT162b2 against hospitalization due to ARI during the Omicron era was 64.1% (95% CI 41.4, 78.0) within 6 months of vaccination and 55.7% (95% CI 25.9, 73.5) ≥ 6 months after vaccination. More detailed temporal analyses of VE (Supplementary Table S1) were limited by the sample size. The effectiveness of hybrid immunity (vaccination plus a reported history of SARS-CoV-2 infection) was 82.4% (95% CI 66.2, 90.8) following the primary series and 77.9% (95% CI 51.6, 89.9) following ≥1 booster (Supplementary Table S2).

The adjusted overall VE of the primary series for African American/Black participants was 64.0% (95% CI 49.9, 74.1) and 82.7% (95% CI 71.9, 89.4) in those who received ≥1 booster, higher than seen in the entire cohort. When the analysis was limited to the period of Omicron predominance, VE of primary series (40.1%, 95% CI 9.2, 60.5) and ≥1 booster doses (67.1%, 95% CI 44.1, 80.7) decreased in African American/Black individuals but remained higher than the overall cohort (Table 4, Supplementary Table S3).

## 4. Discussion

In this test-negative case–control study conducted in a U.S. population of predominantly African American adults, we determined that being vaccinated with a primary series of BNT162b2 resulted in a VE of 58.5% (95% CI 46.0, 68.1) against COVID-19-associated ARI hospitalization in adults from May 2021 to January 2023. In comparison, receipt of ≥1 BNT162b2 booster yielded a VE of 78.9% (95% CI 70.0, 85.1). When analysis was limited to the period of Omicron predominance, VE of the primary series decreased (24.5%, 95% CI −4.5, 45.4), while VE of ≥1 booster was maintained at 60.9% (95% CI 42.0, 73.6). Overall, both the primary series and booster provided protection against COVID-19-associated ARI hospitalizations lasting ≥6 months.

Our study population was comprised of a majority (65.8%) of African American participants who enrolled at two hospitals in metropolitan Atlanta, Georgia. Reassuringly, the adjusted overall VE of a primary series and booster COVID-19 vaccination in this population was similar to what was observed in the larger cohort. Despite a higher vulnerability to severe COVID-19 outcomes among African American, Native Indian, Alaska Native, and Hispanic populations [10,15,16], these populations are underrepresented in the majority of COVID-19 VE studies. To our knowledge, this study is the only VE study conducted in a predominantly African American population and shows consistency with other VE reports of other racial diversity. These data provide reassurance of the benefits of COVID-19 vaccination among African Americans and can help instill confidence to ensure vaccine equity in this population.

Overall, our study was consistent with existing literature in showing that vaccination with BNT162b2 reduces the risk of COVID-19-related hospitalization [11,17,18,19]. A study conducted in the United Kingdom estimated that 2–4 weeks after a second dose, the BNT162b2 vaccine was 90.9% (95% CI: 89.6, 92.0) effective against symptomatic disease caused by the Delta variant but only 65.5% (95% CI: 63.9, 67.0) effective against symptomatic disease caused by the initial Omicron variant [20]. The same study also showed that VE is impacted by the number of doses of BNT162b2 received, with additional doses correlating with higher VE. While VE against symptomatic infection has been shown to decline gradually over several months, VE against hospitalization is higher, more durable, and maintained for ≥6 months in prior studies [10]. Nevertheless, VE against the Omicron variant and its subvariants (e.g., BA.1, BA.2, BA.4/5) has consistently been reduced compared to previously circulating strains [19,21]. In summary, these studies and others corroborate our findings that VE reduces the risk of hospitalization, that a booster dose increases protection, and that protection against hospitalization is durable over a period of ≥6 months but decreases with the Omicron variant and its sublineages.

Underlying clinical and sociodemographic characteristics differed among cases with SARS-CoV-2 when compared to hospitalized controls with ARI caused by other etiologies. Cases were overall younger, more commonly employed, more commonly traveled within the preceding month, and more commonly had children living in their homes. In terms of underlying comorbidities, cases with SARS-CoV-2 were overall healthier and were more commonly hospitalized during the pre-variant/Delta era. These findings likely represent the greater burden of disease caused by the ancestral SARS-CoV-2 strain and the Delta variant on healthy adults who had exposure-based risk factors, in contrast to the Omicron variant, which disproportionately affected older adults with more underlying comorbidities [22,23].

Overall, in our study population, 43.6% had completed a BNT612b2 primary series, and 17.3% had received ≥1 booster vaccination. Vaccinated participants were older and more commonly had comorbidities compared to unvaccinated participants. As has been reported previously [24], there were disparities in vaccine uptake, with fewer African American and Hispanic participants receiving either a primary series or booster vaccination. Prior studies have found that racial and ethnic disparities in vaccine uptake are often mediated through other sociodemographic factors, including economic conditions and social vulnerability [25,26]. These findings underscore the importance of improving vaccination uptake, particularly through interventions that target the social and economic drivers of health disparities.

Limitations of our study include an enrollment rate of 2763 (53.8%), of whom 1571 (56.9%) qualified for the final analyses, which could have introduced biases into the data based on enrollment or vaccine preference characteristics. Also, while this study provides insights into VE in a predominantly African American population, the findings might not be fully generalizable to other racial or ethnic groups in other geographical areas with different sociodemographic characteristics and virus exposures. The test-negative design has some limitations, including selection bias in standard-of-care testing and potential misclassification of COVID-19 status. Despite adjusting for potential confounders, there remains the possibility of unmeasured confounding, including socioeconomic factors, access to healthcare, and compliance with public health measures, which could affect vaccine uptake and exposure risk. This study was insufficiently powered to determine the VE of variant-containing booster vaccinations and variant-specific VEs. This study also pre-dated the circulation of newer Omicron subvariants, including XBB.1.5. Future studies are needed to evaluate the VE of variant-containing booster vaccines.

## 5. Conclusions

In conclusion, we found the BNT162b2 vaccine to be effective in preventing COVID-19-related ARI hospitalizations in a unique population of predominantly African American adults. Reporting VE in racially diverse populations may provide additional reassurance on the protective effects of vaccination and improve equity. VE was greatest following ≥1 booster dose and was durable but decreased during Omicron variant predominance. There is an ongoing need to conduct real-world VE studies as new variants emerge and as population immunity evolves over time to inform public policy decisions.

## Figures and Tables

**Table 1:** Demographic Characteristics of Study Participants by SARS-CoV-2 Infection and Vaccination Status.

		Controls: SARS-CoV-2 Negative (n = 926)	Cases: SARS-CoV-2 Positive (n = 645)	*p*-Value	Never Vaccinated for COVID-19(n = 886)	Primary Series Vaccine (n = 412)	*p*-Value	Primary Series + ≥1 Booster (n = 273)	*p*-Value(vs. Never Vaccinated)
**Age,** **median (IQR), y**		60 [47, 70]	54 [41, 66]	***0.0001***	52 [38, 62]	63 [53, 72]	***<0.0001***	68 [58, 76]	***<0.0001***
**Age by category, No. (%), y**				***0.001***			***<0.0001***		***<0.0001***
	18–49	269 (29.0)	244 (37.8)		398 (44.9)	85 (20.6)		30 (11.0)	
	50–64	315 (34.0)	214 (33.2)		312 (35.2)	138 (33.5)		79 (28.9)	
	65–74	191 (20.6)	108 (16.7)		116 (13.1)	100 (24.3)		83 (30.4)	
	≥75	151 (16.3)	79 (12.2)		60 (6.8)	89 (21.6)		81 (29.7)	
**Female sex,** **No. (%)**		517 (55.8)	372 (57.7)	0.4685	519 (58.6)	219 (53.2)	0.0664	151 (55.3)	0.3393
**Race,** **No. (%)**				0.1973			***<0.0001***		***<0.0001***
	Black	595 (64.3)	439 (68.1)		644 (72.7)	246 (59.7)		144 (52.7)	
	White	212 (22.9)	147 (22.8)		158 (17.8)	119 (28.9)		82 (30.0)	
	Multiracial	24 (2.6)	15 (2.3)		21 (2.4)	7 (1.7)		11 (4.0)	
	Other	15 (1.6)	7 (1.1)		12 (1.4)	7 (1.7)		3 (1.1)	
	Not Specified	80 (8.6)	37 (5.7)		51 (5.8)	33 (8.0)		33 (12.1)	
**Ethnicity, No. (%)**				***0.0467***			***0.0341***		***0.0005***
	Non-Hispanic	787 (85.0)	571 (88.5)		773 (87.2)	362 (87.9)		223 (81.7)	
	Hispanic	32 (3.5)	24 (3.7)		41 (4.6)	8 (1.9)		7 (2.5)	
	Not Specified	107 (11.6)	50 (7.8)		72 (8.1)	42 (10.2)		43 (15.8)	
**Insurance, No. (%)**				**<0.0001**			**<0.0001**		**<0.0001**
	Public	405 (43.7)	257 (39.8)		380 (42.9)	186 (45.1)		96 (35.2)	
	Private	174 (18.8)	217 (33.6)		243 (27.4)	98 (23.8)		50 (18.3)	
	Multiple	258 (27.9)	109 (16.9)		144 (16.3)	105 (25.5)		118 (43.2)	
	None	87 (9.4)	61 (9.5)		117 (13.2)	23 (5.6)		8 (2.9)	
	Other/Unknown	2 (0.2)	1 (0.2)		2 (0.2)	0 (0.0)		1 (0.4)	
**Site of Enrollment, No. (%)**				***0.0093***			***0.0004***		***0.0001***
	#1	405 (43.7)	325 (50.4)		367 (41.4)	214 (51.9)		149 (54.6)	
	#2	521 (56.3)	320 (49.6)		519 (58.6)	198 (48.1)		124 (45.4)	
**Month of Enrollment, No. (%)**				**<0.0001**			**<0.0001**		**<0.0001**
	January–March	178 (19.2)	128 (19.8)		155 (17.5)	95 (23.1)		56 (20.5)	
	April–June	151 (16.3)	86 (13.3)		141 (15.9)	68 (16.5)		28 (10.3)	
	July–September	293 (23.1)	293 (45.4)		344 (38.8)	96 (23.3)		67 (24.5)	
	October–December	383 (41.4)	138 (21.4)		246 (27.8)	153 (37.1)		122 (44.7)	
**Variant Period, No. (%)**				**<0.0001**			**<0.0001**		**<0.0001**
	Pre-Delta/Delta	191 (20.6)	340 (52.7)		429 (48.4)	98 (23.8)		4 (1.5)	
	Omicron	735 (79.4)	305 (47.3)		457 (51.6)	314 (76.2)		269 (98.5)	
**Education level, No. (%)**				***<0.0001***			***0.0015***		***<0.0001***
	<High School Grad/GED	127 (13.7)	65 (10.1)		124 (14.0)	43 (10.4)		25 (9.2)	
	High School Grad/GED	283 (30.6)	162 (25.1)		276 (31.2)	106 (25.7)		63 (23.1)	
	Some College/Associate degree	203 (21.9)	183 (28.4)		226 (25.5)	101 (24.5)		59 (21.6)	
	College Graduate	157 (17.0)	145 (22.5)		158 (17.8)	87 (21.1)		57 (20.9)	
	Advanced Degree	73 (7.9)	54 (8.4)		47 (5.3)	44 (10.7)		36 (13.2)	
	Unknown	83 (9.0)	36 (5.6)		55 (6.2)	31 (7.5)		33 (12.1)	
**Employed, No. (%)**				***<0.0001***			***0.0028***		***<0.0001***
	Yes	198 (21.4)	274 (42.5)		313 (35.3)	107 (26.0)		52 (19.0)	
	No	424 (45.8)	223 (34.6)		329 (37.1)	184 (44.7)		134 (49.1)	
	N/A (Disability)	228 (24.6)	120 (18.6)		201 (22.7)	92 (22.3)		55 (20.1)	
	Unknown	76 (8.2)	28 (4.3)		43 (4.9)	29 (7.0)		32 (11.7)	
**Employed as HCW, No. (%)**				***<0.0001***			0.2671		***0.0003***
	Yes	19 (2.1)	32 (5.0)		29 (3.3)	12 (2.9)		10 (3.7)	
	No	831 (89.7)	585 (90.7)		814 (91.9)	371 (90.0)		231 (84.6)	
	Unknown	76 (8.2)	28 (4.3)		43 (4.9)	29 (7.0)		32 (11.7)	
**Retired, No. (%)**				***<0.0001***			***<0.0001***		***<0.0001***
	Yes	273 (29.5)	138 (21.4)		148 (16.7)	143 (34.7)		120 (44.0)	
	No	347 (37.5)	362 (56.1)		493 (55.6)	148 (35.9)		68 (24.9)	
	N/A (Disability)	230 (24.8)	117 (18.1)		202 (22.8)	92 (22.3)		53 (19.4)	
	Unknown	76 (8.2)	28 (4.3)		43 (4.9)	29 (7.0)		32 (11.7)	
**Residence, No. (%)**				***0.0113***			***0.0034***		***<0.0001***
	Private Home	788 (85.1)	576 (89.3)		791 (89.3)	356 (86.4)		217 (79.5)	
	SNF/Assisted Living/LTCF	24 (2.6)	14 (2.2)		8 (0.9)	13 (3.2)		17 (6.2)	
	Other	33 (3.6)	26 (4.0)		41 (4.6)	12 (2.9)		6 (2.2)	
	Unknown	81 (8.7)	29 (4.5)		46 (5.2)	31 (7.5)		33 (12.1)	

Abbreviations: GED = General Educational Development; IQR = Interquartile Range; HCW = Healthcare Worker; SNF = Skilled Nursing Facility; LTCF = Long-term Care Facility.

**Table 2:** Social and Behavioral Risk Factors of Study Participants by SARS-CoV-2 Infection and Vaccination Status.

		Controls: SARS-CoV-2 Negative (n = 926)	Cases: SARS-CoV-2 Positive (n = 645)	*p*-Value	Never Vaccinated for COVID-19 (n = 886)	Primary Series Vaccine (n = 412)	*p*-Value	Primary Series + ≥1 Booster (n = 273)	*p*-Value(vs. Never Vaccinated)
**Any Children age < 18 at home, No. (%)**				**<0.0001**			**<0.0001**		**<0.0001**
	None	654 (70.6)	408 (63.3)		543 (61.3)	315 (76.5)		204 (74.7)	
	≥1	196 (21.2)	208 (32.2)		300 (33.9)	67 (16.3)		37 (13.6)	
	Unknown	76 (8.2)	29 (4.5)		43 (4.9)	30 (7.3)		32 (11.7)	
**Children aged < 5 y at home, No. (%)**				**0.0006**			**<0.0001**		**<0.0001**
	None	763 (82.4)	526 (81.6)		707 (79.8)	358 (86.9)		224 (82.1)	
	≥1	87 (9.4)	90 (14.0)		136 (15.3)	24 (5.8)		17 (6.2)	
	Unknown	76 (8.2)	29 (4.5)		43 (4.9)	30 (7.3)		32 (11.7)	
**Children aged 5–17 y at home, No. (%)**				**<0.0001**			**<0.0001**		**<0.0001**
	None	689 (74.4)	451 (69.9)		599 (67.6)	328 (79.6)		213 (78.0)	
	≥1	161 (17.4)	165 (25.6)		244 (27.5)	54 (13.1)		28 (10.3)	
	Unknown	76 (8.2)	29 (4.5)		43 (4.9)	30 (7.3)		32 (11.7)	
**Adherence to Social Distancing, No. (%)**				**<0.0001**			0.2269		**0.0004**
	Always	518 (55.9)	410 (63.6)		515 (58.1)	250 (60.7)		163 (59.7)	
	Usually	139 (15.0)	120 (18.6)		155 (17.5)	67 (16.3)		37 (13.6)	
	About half the time	91 (9.8)	45 (7.0)		79 (8.9)	33 (8.0)		24 (8.8)	
	Seldom/Never	101 (10.9)	41 (6.4)		93 (10.5)	32 (7.8)		17 (6.2)	
	Unknown	77 (8.3)	29 (4.5)		44 (5.0)	30 (7.3)		32 (11.7)	
**Adherence to Masking in Public (indoors), No. (%)**				**<0.0001**			0.1192		**0.0007**
	Always	516 (55.7)	435 (67.4)		533 (60.2)	253 (61.4)		165 (60.4)	
	Usually	123 (13.3)	90 (14.0)		131 (14.8)	52 (12.6)		30 (11.0)	
	About half the time	72 (7.8)	42 (6.5)		59 (6.7)	35 (8.5)		20 (7.3)	
	Seldom/Never	138 (14.9)	49 (7.6)		119 (13.4)	42 (10.2)		26 (9.5)	
	Unknown	77 (8.3)	29 (4.5)		44 (5.0)	30 (7.3)		32 (11.7)	
**Adherence to Masking in Public (outdoors), No. (%)**				**<0.0001**			0.4898		**0.0002**
	Always	419 (45.2)	365 (56.6)		463 (52.3)	201 (48.8)		120 (44.0)	
	Usually	93 (10.0)	82 (12.7)		103 (11.6)	49 (11.9)		23 (8.4)	
	About half the time	80 (8.6)	52 (8.1)		69 (7.8)	34 (8.3)		29 (10.6)	
	Seldom/Never	257 (27.8)	117 (18.1)		207 (23.4)	98 (23.8)		69 (25.3)	
	Unknown	77 (8.3)	29 (4.5)		44 (5.0)	30 (7.3)		32 (11.7)	
**Out-of-state travel in the past month, No. (%)**				**<0.0001**			0.1954		**0.0004**
	None	770 (83.2)	504 (78.1)		731 (82.5)	337 (81.8)		206 (75.5)	
	Domestic/International	79 (8.5)	112 (17.4)		111 (12.5)	45 (10.9)		35 (12.8)	
	Unknown	77 (8.3)	29 (4.5)		44 (5.0)	30 (7.3)		32 (11.7)	
**Smoking, No. (%)**				**<0.0001**			0.2785		**0.0077**
	No	388 (41.9)	383 (59.4)		445 (50.2)	200 (48.5)		126 (46.2)	
	Yes (any)	455 (49.1)	224 (34.7)		387 (43.7)	177 (43.0)		115 (42.1)	
	Unknown	83 (9.0)	38 (5.9)		54 (6.1)	35 (8.5)		32 (11.7)	
**Drink Alcohol, No. (%)**				**0.0109**			0.192		**<0.0001**
	No	543 (58.6)	381 (59.1)		519 (58.6)	240 (58.3)		165 (60.4)	
	Yes	307 (33.1)	235 (36.4)		324 (36.6)	142 (34.5)		76 (27.8)	
	Unknown	76 (8.2)	29 (4.5)		43 (4.9)	30 (7.3)		32 (11.7)	

**Table 3:** Clinical Features and Outcomes of Study Participants by SARS-CoV-2 Infection and Vaccination Status.

		Controls: SARS-CoV-2 Negative (n = 926)	Cases: SARS-CoV-2 Positive (n = 645)	*p*-Value	Never Vaccinated for COVID-19 (n = 886)	Primary Series Vaccine (n = 412)	*p*-Value	Primary Series + ≥1 Booster(n = 273)	*p*-Value(vs. Never Vaccinated)
**BMI, median (IQR)**		28.1 [23.0, 35.1]	29.9 [24.9, 36.5]	**0.0224**	29.3 [23.8, 36.3]	28.0 [23.2, 35.0]	0.0640	28.2 [23.7, 34.7]	0.0960
BMI by category * **, No. (%)**				**<0.0001**			**0.0445**		0.2959
	Underweight (<18.5)	55 (5.9)	14 (2.2)		34 (3.8)	27 (6.6)		8 (2.9)	
	Healthy (18.5–24.9)	261 (28.2)	149 (23.1)		227 (25.6)	106 (25.7)		77 (28.2)	
	Overweight (25.0–29.9)	232 (25.1)	156 (24.2)		205 (23.1)	106 (25.7)		77 (28.2)	
	Obese, class 1 (30.0–34.9)	141 (15.2)	121 (18.8)		153 (17.3)	68 (16.5)		41 (15.0)	
	Obese, class 2–3 (≥35.0)	232 (25.1)	185 (28.7)		247 (27.9)	103 (25.0)		67 (24.5)	
	Unknown	5 (0.5)	20 (3.1)		20 (2.3)	2 (0.5)		3 (1.1)	
**Chronic Conditions, No. (%)**									
	Chronic Respiratory Disease	392 (42.3)	161 (25.0)	**<0.0001**	280 (31.6)	168 (40.8)	**0.0012**	105 (38.5)	**0.0354**
	Chronic Obstructive Pulmonary Disease	240 (25.9)	74 (11.5)	**<0.0001**	140 (15.8)	103 (25.0)	**<0.0001**	71 (26.0)	**0.0001**
	Asthma/Reactive Airway Disease	168 (18.1)	103 (16.0)	0.2620	152 (17.2)	69 (16.7)	0.8555	50 (18.3)	0.6589
	Use oxygen supplementation at home	129 (13.9)	26 (4.0)	**<0.0001**	67 (7.6)	52 (12.6)	**0.0033**	36 (13.2)	**0.0043**
	Blood Disorder (e.g., Sickle cell disease)	135 (14.6)	68 (10.5)	**0.0190**	96 (10.8)	85 (20.6)	**<0.0001**	22 (8.1)	0.1847
	Cardiac Disease	663 (71.6)	375 (58.1)	**<0.0001**	502 (56.7)	312 (75.7)	**<0.0001**	224 (82.1)	**<0.0001**
	Arrhythmia	158 (17.1)	63 (9.8)	**<0.0001**	89 (10.0)	77 (18.7)	**<0.0001**	55 (20.1)	**<0.0001**
	Coronary Artery Disease	139 (15.0)	66 (10.2)	**0.0057**	90 (10.2)	60 (14.6)	**0.0209**	55 (20.1)	**<0.0001**
	Congestive Heart Failure (CHF)	269 (29.0)	104 (16.1)	**<0.0001**	180 (20.3)	109 (26.5)	**0.0133**	84 (30.8)	**0.0003**
	Hypertension	598 (64.6)	332 (51.5)	**<0.0001**	450 (50.8)	275 (66.7)	**<0.0001**	205 (75.1)	**<0.0001**
	Peripheral Vascular Disease (PVD)	34 (3.7)	14 (2.2)	0.0890	19 (2.1)	11 (2.7)	0.5576	18 (6.6)	**0.0003**
	Chronic Kidney Disease	222 (24.0)	122 (18.9)	**0.0171**	147 (16.6)	109 (26.5)	**<0.0001**	88 (32.2)	**<0.0001**
	Chronic Liver Disease	17 (1.8)	8 (1.2)	0.4164	12 (1.4)	4 (1.0)	0.7878	9 (3.3)	0.0639
	Cystic Fibrosis	10 (1.1)	2 (0.3)	0.1380	5 (0.6)	4 (1.0)	0.4765	3 (1.1)	0.4012
	Dementia	24 (2.6)	16 (2.5)	0.8905	12 (1.4)	12 (2.9)	0.0524	16 (5.9)	**<0.0001**
	Diabetes Mellitus	282 (30.5)	166 (25.7)	**0.0416**	224 (25.3)	123 (29.9)	0.0832	101 (37.0)	**0.0002**
	Seizure Disorder	29 (3.1)	12 (1.9)	0.1200	21 (2.4)	11 (2.7)	0.7459	9 (3.3)	0.3878
	Any immunosuppressive condition (incl. cancer, HIV)	333 (36.0)	162 (25.1)	**<0.0001**	254 (28.7)	172 (41.7)	**<0.0001**	69 (25.3)	0.2742
	Cancer	160 (17.3)	53 (8.2)	**<0.0001**	86 (9.7)	90 (21.8)	**<0.0001**	37 (13.6)	0.0712
	Chronic steroid use	111 (12.0)	76 (11.8)	0.9022	100 (11.3)	71 (17.2)	**0.0032**	16 (5.9)	**0.0090**
	HIV/AIDS	50 (5.4)	22 (3.4)	0.0637	51 (5.8)	16 (3.9)	0.1558	5 (1.8)	**0.0058**
	Solid Organ Transplant	42 (4.5)	46 (7.1)	**0.0277**	34 (3.8)	36 (8.7)	**0.0003**	18 (6.6)	**0.0544**
	Hematopoietic Cell Transplant	4 (0.4)	1 (0.2)	0.6543	1 (0.1)	2 (0.5)	0.2381	2 (0.7)	0.1401
	Splenectomy	1 (0.1)	0 (0.0)	1.0000	0 (0.0)	0 (0.0)	--	1 (0.4)	0.2355
	Rheumatologic/Connective Tissue Disease	71 (7.7)	34 (5.3)	0.0614	60 (6.8)	27 (6.6)	0.8834	18 (6.6)	0.9180
	Stroke	81 (8.7)	40 (6.2)	0.0626	56 (6.3)	34 (8.3)	0.2022	31 (11.4)	**0.0058**
	None	56 (6.1)	134 (20.8)	**<0.0001**	156 (17.6)	22 (5.3)	**<0.0001**	12 (4.4)	**<0.0001**
**Pregnant**		19 (2.1)	21 (3.3)	0.1361	32 (3.6)	7 (1.7)	0.0791	1 (0.4)	**0.0027**
Charlson Comorbidity Index **, median (IQR)									
	Updated (uCCI) (range, 0–14)	1 [0, 3]	1 [0, 2]	**<0.0001**	1 [0, 2]	1 [0, 3]	**<0.0001**	2 [1, 3]	**<0.0001**
	Classical (cCCI) (range, 0–15)	2 [1, 3]	1 [0, 2]	**<0.0001**	1 [0, 3]	2 [1, 3]	**0.0002**	2 [1, 3]	**0.0003**
**Prior SARS-CoV-2 Infection, No. (%)**				**<0.0001**			0.1002		0.3612
	Yes	166 (17.9)	56 (8.7)		114 (12.9)	67 (16.3)		41 (15.0)	
	No	760 (82.1)	589 (91.3)		772 (87.1)	345 (83.7)		232 (85.0)	
**Hospital History, No. (%)**									
	Admitted to ICU	229 (24.7)	142 (22.0)	0.2127	203 (22.9)	106 (25.7)	0.2675	62 (22.7)	0.9448
	Ventilator Support	181 (19.5)	118 (18.3)	0.5341	168 (19.0)	86 (20.9)	0.4189	45 (16.5)	0.3553
**Hospital Discharge:**				0.4129			**0.0071**		**0.0013**
	Discharged to home	804 (86.8)	561 (87.0)		773 (87.2)	361 (87.6)		231 (84.6)	
	Discharged to assisted living	39 (4.2)	28 (4.3)		32 (3.6)	15 (3.6)		20 (7.3)	
	Discharged to another hospital	10 (1.1)	4 (0.6)		7 (0.8)	3 (0.7)		4 (1.5)	
	Discharged to hospice	32 (3.5)	14 (2.2)		17 (1.9)	18 (4.4)		11 (4.0)	
	Death	20 (2.2)	21 (3.3)		23 (2.6)	12 (2.9)		6 (2.2)	
	Other/Unknown	21 (2.3)	17 (2.6)		34 (3.8)	3 (0.7)		1 (0.4)	

* BMI data was missing for 25 participants. ** Charlson comorbidity index definitions and weights used were applied as previously described, with exception of asymptomatic HIV/AIDS inclusion in the cCCI and uremia as a consideration for renal disease. Abbreviations: AIDS, acquired immunodeficiency syndrome. BMI, body mass index. HIV, human immunodeficiency virus.

**Table 4:** BNT162b2 vaccine effectiveness against COVID-19-associated hospitalization for acute respiratory infection among adults with a SARS-CoV-2 test.

	Pre/Delta Era (2 May 2020–19 December 2021)		Omicron Era (20 December 2021–31 January 2023)		Overall	
**All Participants**	**Unadjusted (95% CI)**	**Adjusted (95% CI)**	**Unadjusted (95% CI)**	**Adjusted (95% CI)**	**Unadjusted (95% CI)**	**Adjusted (95% CI)**
**Primary Series**						
** Overall**	79.6 (67.4, 87.2)	80.8 (67.6, 88.7)	19.6 (−9.6, 41.0)	24.5 (−4.5, 45.4)	59.7 (48.4, 68.6)	58.5 (46.0, 68.1)
** <6 months since completion**	84.1 (71.8, 91.0)	84.4 (70.7, 91.6)	−12.2 (−87.3, 32.8)	−8.9 (−90.0, 37.6)	55.3 (34.8, 69.3)	54.7 (32.4, 69.6)
** ≥6 months since completion**	66.9 (30.8, 84.2)	74.7 (44.5, 88.4)	27.9 (−1.5, 48.8)	34.0 (5.6, 53.8)	61.9 (49.0, 71.6)	61.5 (47.7, 71.7)
**Booster + (Primary series + ≥1 booster)**						
** Overall**	86.6 (−30.1, 98.6)	89.9 (0.1, 99.0)	50.9 (30.0, 65.5)	60.9 (42.0, 73.6)	76.4 (67.5, 82.9)	78.9 (70.0, 85.1)
** <6 months since completion**	86.6 (−30.1, 98.6)	89.9 (0.1, 99.0)	55.8 (30.4, 71.9)	64.1 (41.4, 78.0)	78.6 (67.3, 86.0)	79.3 (67.4, 86.8)
** ≥6 months since completion**	--	--	44.5 (11.2, 65.3)	55.7 (25.9, 73.5)	73.4 (58.5, 83.1)	77.8 (64.2, 86.2)
	**Pre/Delta Era**		**Omicron Era**		**Overall**	
**African American/Black race (Any)**	**Unadjusted (95% CI)**	**Adjusted (95% CI)**	**Unadjusted (95% CI)**	**Adjusted (95% CI)**	**Unadjusted (95% CI)**	**Adjusted (95% CI)**
**Primary Series**						
** Overall**	75.1 (55.7, 86.0)	75.1 (53.6, 86.7)	35.9 (4.3, 57.0)	39.6 (8.6, 60.1)	65.2 (52.3, 74.6)	63.8 (49.8, 74.0)
** <6 months since completion**	79.7 (58.7, 90.0)	79.2 (55.4, 90.3)	−2.7 (−90.7, 44.7)	1.9 (−85.7, 49.3)	53.6 (26.4, 70.8)	48.9 (17.1, 68.5)
** ≥6 months since completion**	64.6 (13.7, 85.4)	69.9 (23.9, 88.1)	47.7 (16.7, 67.2)	50.7 (20.8, 69.4)	70.8 (56.9, 80.3)	70.6 (56.1, 80.3)
**Booster + (Primary series + ≥1 booster)**						
** Overall**	-	-	60.8 (36.1, 75.9)	68.5 (46.5, 81.5)	82.4 (72.2, 88.8)	83.4 (73.1, 89.8)
** <6 months since completion**	-	-	71.6 (44.3, 85.5)	75.8 (50.8, 88.1)	87.4 (75.9, 93.4)	86.9 (74.4, 93.3)
** ≥6 months since completion**	-	-	43.9 (−6.3, 70.4)	59.5 (19.7, 79.6)	74.2 (52.1, 86.0)	78.9 (60.0, 88.9)
**Not-African American/Black race**	**Unadjusted (95% CI)**	**Adjusted (95% CI)**	**Unadjusted (95% CI)**	**Adjusted (95% CI)**	**Unadjusted (95% CI)**	**Adjusted (95% CI)**
**Primary Series**						
** Overall**	84.7 (64.8, 93.4)	91.0 (75.0, 96.8)	−11.3 (−85.1, 33.1)	−14.8 (−95.1, 32.4)	46.9 (19.9, 64.8)	48.8 (13.0, 63.7)
** <6 months since completion**	88.4 (68.8, 95.7)	93.2 (77.9, 97.9)	−40.0 (−244.0, 45.5)	−38.1 (−275.7, 49.3)	56.2 (15.7, 77.2)	52.7 (6.5, 76.0)
** ≥6 months since completion**	70.5 (−12.1, 92.2)	85.4 (32.6, 96.8)	−6.3 (−82.1, 37.9)	−6.5 (−86.2, 39.0)	42.4 (8.5, 63.7)	40.1 (2.3, 63.3)
**Booster + (Primary series + ≥1 booster)**						
** Overall**	-	-	36.9 (−8.6, 63.3)	48.1 (5.9, 71.3)	65.3 (44.0, 78.5)	71.4 (51.4, 83.2)
** <6 months since completion**	-	-	30.0 (−35.2, 63.7)	42.6 (−15.9, 71.5)	60.6 (28.2, 78.3)	65.2 (33.6, 81.7)
** ≥6 months since completion**	-	-	44.1 (−13.8, 72.5)	51.2 (−6.2, 77.6)	70.3 (42.1, 84.7)	76.8 (52.4, 88.7)

## Data Availability

The data supporting the conclusions of this article will be made available by the authors upon reasonable request.

## Supplementary Materials

The following supporting information can be downloaded at: https://www.mdpi.com/article/10.3390/vaccines12060657/s1, Supplementary Results; Supplementary Table S1. BNT162b2 vaccine effectiveness against COVID-19-associated hospitalization for acute respiratory infection stratified by time since vaccination; Supplementary Table S2. Effectiveness of hybrid immunity, defined as BNT162b2 vaccination plus self-reported history of SARS-CoV-2 infection; Supplementary Table S3. Sensitivity analysis for BNT162b2 Vaccine effectiveness against COVID-19-associated hospitalization for acute respiratory infection among adults who identified as Black race (only) and White race (only).
