# Supplementary material for: Effectiveness of BNT162b2 Vaccine for Preventing COVID-19-Related Hospitalizations: A Test-Negative Case–Control Study
Source: Vaccines (Basel). 2024 Jun 13;12(6):657. doi: 10.3390/vaccines12060657 (PMC11209557; doi:10.3390/vaccines12060657)
Supplement: Supplementary file 1 [file vaccines-12-00657-s001.zip › vaccines-3003313-supplementary.pdf]

SUPPLEMENTARY MATERIAL

Supplementary Results

Study Population

Among 5139 potentially eligible adults, 2763 (53.8%) were enrolled, of whom 1571 (56.9%) were eligible for inclusion in the BNT162b2 analysis (**Figure 1**). Of the 2376 (46.2%) not enrolled, the most common reasons for non-enrollment were refusal by patient or LAR (64.6%) or discharge prior to being approached by study staff (23.8%). Overall 1192 (43.1%) patients were excluded from the analyses for one of the following reasons: 187 (7.3%) did not have any vaccination records available, 656 (55.0%) received a SARS-CoV-2 vaccine other than BNT162b2, 45 (3.8%) received fewer than the number of BNT162b2 doses required for eligibility, 247 (20.7%) received the most recent vaccine <14 days before hospital admission, and 57 (4.8%) were missing COVID-19 test results.

Vaccine Effectiveness of Bivalent Boosters

When the vaccine effectiveness analysis was stratified to participants who had received a bivalent BNT162b2 booster during the study period, the crude VE of a bivalent booster was 83.0% (95% CI 63.4, 92.1). However, when this analysis was limited to those who were enrolled after September 1, 2022 (when the bivalent booster became available), the crude VE decreased with widened confidence intervals (14.3%, 95% CI -95.2, 62.4), likely reflective of inadequate statistical power.

Supplementary Table S1. BNT162b2 Vaccine effectiveness against COVID-19-associated hospitalization for acute respiratory infection stratified by *time since vaccination*.

	**Pre/Delta Era (May 2, 2020-Dec 19, 2021)**		**Omicron Era (Dec 20, 2021-Jan 31, 2023)**		**Overall**	
**All Participants**	**Unadjusted (95% CI)**	**Adjusted (95% CI)**	**Unadjusted (95% CI)**	**Adjusted (95% CI)**	**Unadjusted (95% CI)**	**Adjusted (95% CI)**
**Primary Series**						
Overall	79.6 (67.4, 87.2)	80.8 (67.6, 88.7)	19.6 (-9.6, 41.0)	24.5 (-4.5, 45.4)	59.7 (48.4, 68.6)	58.5 (46.0, 68.1)
0-3 months since completion	91.7 (80.8, 96.4)	89.8 (75.0, 95.8)	26.0 (-48.0, 63.0)	26.8 (-52.2, 64.8)	72.9 (62.5, 80.4)	70.9 (49.3, 83.3)
4-6 months since completion	59.8 (21.5, 79.4)	72.0 (42.7, 86.3)	-30.3 (-158.2, 34.3)	-22.0 (-151.8, 40.8)	24.2 (-21.7, 52.8)	26.9 (-19.4, 55.3)
7-9 months since completion	74.4 (32.5, 90.3)	81.3 (48.5, 93.2)	-27.4 (-113.3, 23.9)	-25.8 (-113.8, 26.0)	39.7 (5.7, 61.4)	35.3 (-2.8, 59.4)
10-12 months since completion	-	-	45.9 (1.0, 70.5)	50.9 (7.8, 73.8)	74.6 (54.3, 85.9)	71.2 (47.1, 84.3)
>12 months since completion	-	-	39.8 (0.03, 63.7)	49.0 (13.4, 70.0)	71.2 (53.2, 82.3)	72.5 (54.5, 83.4)
**Booster+ (Primary series+≥1 booster)**						
Overall	86.6 (-30.1, 98.6)	89.9 (0.1, 99.0)	50.9 (30.0, 65.5)	60.9 (42.0, 73.6)	76.4 (67.5, 82.9)	78.9 (70.0, 85.1)
0-3 months since completion	86.6 (-30.1, 98.6)	89.9 (0.1, 99.0)	41.9 (4.6, 64.6)	52.3 (19.2, 71.8)	75.3 (63.2, 83.5)	73.0 (55.8, 83.5)
4-6 months since completion	-	-	76.1 (46.3, 89.4)	81.8 (57.7, 92.2)	85.4 (74.8, 91.5)	89.7 (76.7, 95.5)
7-9 months since completion	-	-	18.1 (-54.5, 56.6)	32.7 (-31.8, 65.6)	72.2 (55.3, 82.7)	64.9 (33.0, 81.6)
10-12 months since completion	-	-	71.9 (26.7, 89.2)	76.3 (36.4, 91.2)	86.5 (65.3, 94.8)	87.4 (67.0, 95.2)
>12 months since completion	-	-	23.6 (-147.6, 76.4)	41.6 (-94.9, 82.5)	63.4 (-17.4, 88.6)	69.5 (-1.0, 90.8)

Supplementary Table S2. Effectiveness of hybrid immunity, defined as BNT162b2 vaccination plus self-reported history of SARS-CoV-2 infection.

	**Pre/Delta Era**		**Omicron Era**		**Overall**	
**All Participants**	**Unadjusted (95% CI)**	**Adjusted (95% CI)**	**Unadjusted (95% CI)**	**Adjusted (95% CI)**	**Unadjusted (95% CI)**	**Adjusted (95% CI)**
**Primary Series**						
Overall	95.2 (61.0, 99.4)	96.1 (66.2, 99.5)	59.9 (20.2, 79.9)	62.5 (23.9, 81.5)	82.5 (66.7, 90.8)	82.4 (66.2, 90.8)
<6 months since completion	--	--	48.7 (-89.8, 86.1)	48.3 (-104.3, 86.9)	83.9 (44.0, 95.4)	80.5 (29.6, 94.6)
≥6 months since completion	87.1 (-24.9, 98.7)	91.1 (9.0, 99.1)	63.0 (18.2, 83.3)	64.3 (20.2, 84.0)	81.9 (62.2, 91.3)	82.8 (63.7, 91.9)
**Booster+ (Primary series+≥1 booster)**						
Overall	--	--	51.9 (-3.9, 77.7)	60.1 (10.3, 82.3)	77.4 (52.0, 89.3)	77.9 (51.6, 89.9)
<6 months since completion	--	--	65.8 (-2.2, 88.5)	73.6 (17.9, 91.5)	83.9 (52.5, 94.6)	84.2 (51.7, 94.9)
≥6 months since completion	--	--	28.7 (-106.8, 75.4)	39.6 (-81.8, 79.9)	66.5 (4.0, 88.3)	68.6 (7.5, 89.4)

Supplementary Table S3. Sensitivity analysis for BNT162b2 Vaccine effectiveness against COVID-19-associated hospitalization for acute respiratory infection among adults who identified as Black race (*only*) and White race (*only*).

	**Pre/Delta Era**		**Omicron Era**		**Overall**	
**AA/Black (Only)**	**Unadjusted (95% CI)**	**Adjusted (95% CI)**	**Unadjusted (95% CI)**	**Adjusted (95% CI)**	**Unadjusted (95% CI)**	**Adjusted (95% CI)**
**Primary Series**						
Overall	74.9 (55.3, 85.9)	74.8 (53.0, 86.5)	36.5 (5.0, 57.6)	40.1 (9.2, 60.5)	65.3 (52.4, 74.7)	64.0 (49.9, 74.1)
<6 months since completion	79.6 (58.3, 90.0)	78.9 (54.7, 90.1)	-3.1 (-91.6, 44.5)	-1.5 (-90.6, 49.1)	53.4 (26.0, 70.6)	48.5 (16.5, 68.2)
≥6 months since completion	64.3 (13.0, 85.3)	69.5 (23.0, 87.9)	48.7 (17.9, 68.0)	51.8 (22.0, 70.2)	71.2 (57.3, 80.6)	71.0 (56.6, 80.7)
**Booster+ (Primary series+≥1 booster)**						
Overall	-	-	59.2 (33.5, 75.0)	67.1 (44.1, 80.7)	81.7 (71.0, 88.4)	82.7 (71.9, 89.4)
<6 months since completion	-	-	70.7 (42.4, 85.1)	74.9 (48.9, 87.6)	87.0 (75.1, 93.2)	86.5 (73.5, 93.1)
≥6 months since completion	-	-	41.1 (-11.9, 69.0)	57.2 (15.0, 78.5)	72.9 (49.6, 85.4)	77.7 (57.6, 88.3)
**White (Only)**	**Unadjusted (95% CI)**	**Adjusted (95% CI)**	**Unadjusted (95% CI)**	**Adjusted (95% CI)**	**Unadjusted (95% CI)**	**Adjusted (95% CI)**
**Primary Series**						
Overall	82.5 (58.1, 92.7)	90.6 (71.2, 96.9)	-38.6 (-163.0, 26.9)	-50.3 (-196.1, 23.8)	44.2 (9.4, 65.7)	41.3 (1.4, 65.1)
<6 months since completion	86.9 (63.3, 95.3)	93.1 (75.4, 98.0)	-29.7 (-273.4, 54.9)	-21.8 (-302.4, 63.1)	60.1 (18.2, 80.6)	61.0 (15.2, 82.0)
≥6 months since completion	65.1 (-41.5, 91.4)	84.5 (21.1, 97.0)	-41.1 (-178.3, 28.4)	-50.9 (-207.1, 25.9)	33.0 (-16.7, 61.6)	30.3 (-25.8, 61.3)
**Booster+ (Primary series+≥1 booster)**						
Overall	-	-	28.6 (-41.3, 64.0)	46.6 (-13.6, 74.9)	65.1 (37.8, 80.5)	74.1 (50.6, 86.5)
<6 months since completion	-	-	19.7 (-77.8, 63.7)	44.2 (-35.7, 77.1)	59.7 (18.9, 79.9)	70.2 (34.9, 86.4)
≥6 months since completion	-	-	39.6 (-51.3, 75.9)	44.4 (-48.5, 79.1)	71.8 (34.2, 87.9)	78.3 (46.8, 91.2)
